# Supplementary figures and images for: Nuclear and structural dynamics during the establishment of a specialized effector-secreting cell by Magnaporthe oryzae in living rice cells
Source: BMC Cell Biol. 2017 Jan 26;18:11. doi: 10.1186/s12860-017-0126-z (PMC5270211; doi:10.1186/s12860-017-0126-z)

## Slide 1
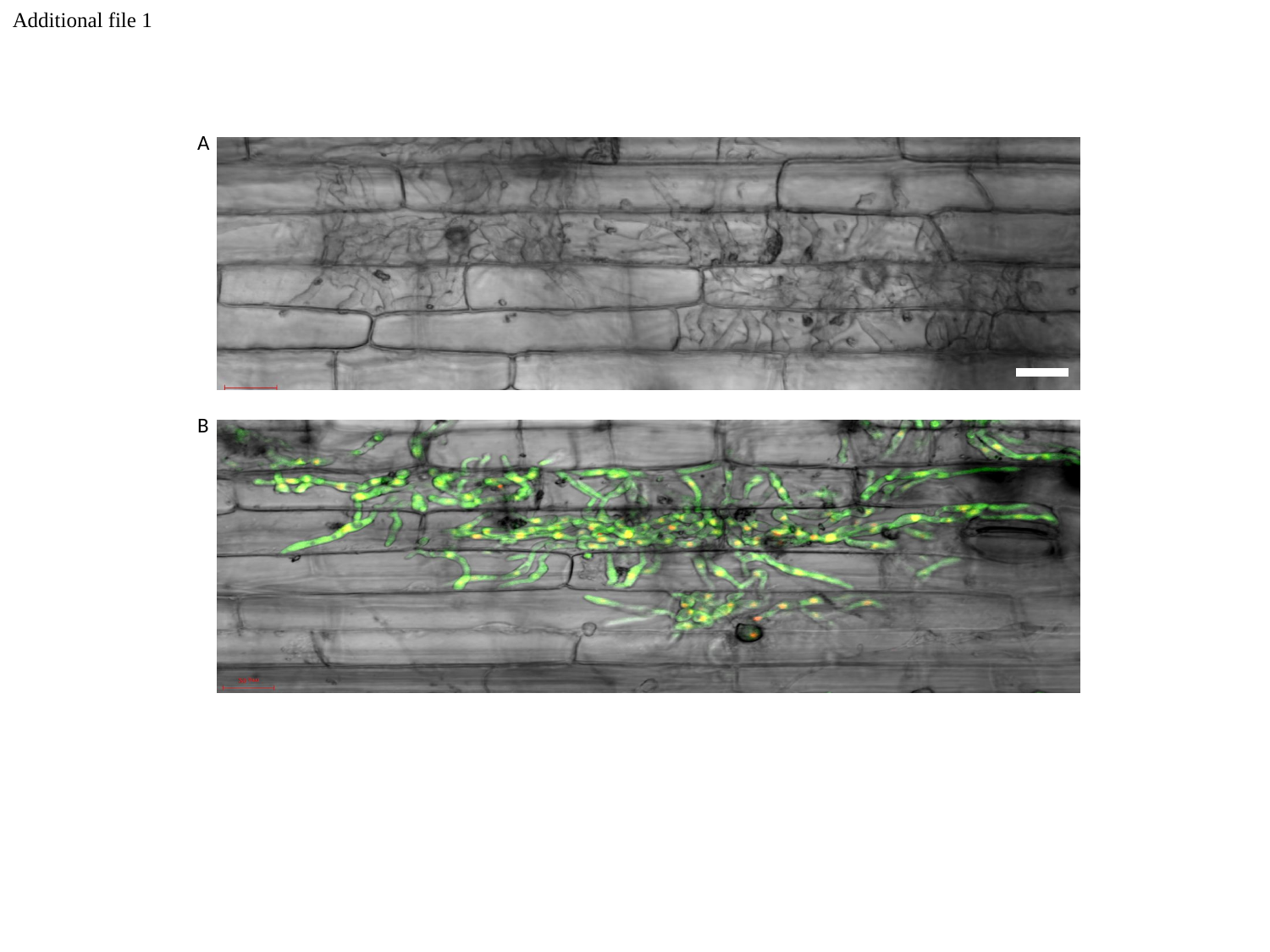

Additional file 1
A
B

Supplement: Additional file 1: — Morphology and pathogenicity of M. oryzae transformant CKF2138 is consistent with wild-type strain. Single plane merged bright-field and fluorescence confocal images of the M. oryzae wild-type strain O-137 (A) and the transformant CKF2138 (B) invading sheath cells of the rice strain YT16 at 48 hpi. M. oryzae CKF2138 constitutively expresses cytoplasmic EYFP (green) and histone H1-tdTomato (red). By this time, both strains have fully colonized initially invaded host cells and have invaded one to two neighboring cells away. The transformant remains consistent in morphology and growth stage with the wild-type strain. Note the advantage of the transformant, whose fluorescent hyphae are more readily distinguished compared to the wild-type hyphae. Bar = 20 μm. (PPTX 1654 kb) [file 12860_2017_126_MOESM1_ESM.pptx]
